# Antibacterial Activity of Palmarosa (*Cymbopogon martini* (Roxb.) Will.Watson) Essential Oil and Geraniol Against Clinical Isolates from Respiratory, Skin, and Soft Tissue Infections

**DOI:** 10.3390/pharmaceutics18010039

**Published:** 2025-12-27

**Authors:** Pilar Cebollada, Elena Alvarado, Cristina Seral, Víctor López

**Affiliations:** 1 Department of Pharmacy, Faculty of Health Sciences, Universidad San Jorge, 50830 Villanueva de Gállego, Zaragoza, Spain; mpcebollada@usj.es; 2 Hospital Clínico Universitario Lozano Blesa, Universidad de Zaragoza, 50009 Zaragoza, Zaragoza, Spaincseral@unizar.es (C.S.); 3 Instituto Agroalimentario de Aragón-IA2, CITA-Universidad de Zaragoza, 50013 Zaragoza, Zaragoza, Spain

**Keywords:** antibacterial activity, antimicrobial resistance, essential oils, geraniol, *Cymbopogon martini*, *Staphylococcus* spp., *Streptococcus* spp.

## Abstract

**Background/Objectives**: Essential oils are liquid natural volatile mixture of compounds with several bioactive properties, which make them useful in a wide range of pharmaceutical applications. The aim of this work is to explore the antimicrobial impact of *Cymbopogon martini* essential oil against human clinical bacterial isolates from the skin and respiratory tract while also assessing its impact on mammalian cells. Geraniol, its main component according to GC-MS analysis, was evaluated under the same conditions. **Methods**: The composition of the essential oil was provided by the supplier. To elucidate the antimicrobial activity, the minimum inhibitory concentration (MIC) and the minimum bactericidal concentration (MBC) were determined. The impact on mammalian hepatic cells was determined using the 3-(4,5-dimethylthiazol-2-yl)-2,5-diphenyltetrazolium bromide (MTT) assay. **Results**: The essential oil showed activity against Gram-positive bacteria from the *Streptococcus* and *Staphylococcus* genera, with MIC values ranging from 125 to 250 µg mL^−1^ for *Streptococcus agalactiae*, *Streptococcus anginosus*, *Streptococcus disgalactiae*， and *Streptococcus pyogenes*. It also displayed activity against some of the tested Gram-negative bacteria, namely, *Escherichia coli* (MIC 350 µg mL^−1^), *Moraxella catarrhalis* (MIC 250 µg mL^−1^), and *Morganella morganii* (MIC 350 µg mL^−1^). In most cases, the essential oil showed lower MIC values than geraniol. Additionally, palmarosa oil had a weaker impact than geraniol in HepG2 cells. **Conclusions**: Both the essential oil and the pure compound exhibited activity against clinical isolates obtained from skin and respiratory tract samples.

## 1. Introduction

Essential oils are natural products obtained by steam distillation, mechanical pressing of citrus rinds, or dry distillation from aromatic plants, characterized by the occurrence of diverse volatile compounds, mainly aliphatic and cyclic hydrocarbons belonging to the monoterpenoid class [1]. Recent studies have highlighted the bioactive properties of these products, namely, their anti-inflammatory, antibacterial, and antifungal properties [2,3,4]. Only a fraction of known plant species are classified as aromatic, and they are mainly concentrated within certain botanical families such as the Lamiaceae, Rutaceae, Myrtaceae, or Poaceae [5].

Species from the *Cymbopogon* genus (Poaceae) have a long history in traditional medicine across Asia, South America, and Africa, where the leaves and other parts of the plant are used for herbal teas or decoctions. Traditionally, plants from this genus have been employed to treat conditions such as fevers, respiratory tract infections, or digestive and inflammatory disorders [6]. *Cymbopogon martini* (*C. martini*), commonly known as palmarosa, is cultivated in India for its essential oil and is rich in monoterpenes and monoterpenic alcohols such as geraniol, which represents around 75–80% of its composition [7]. Palmarosa essential oil has been extensively used in ancient Indian and Southeast Asian traditional medicines and has been studied in recent years, showing noteworthy biological properties [8,9,10,11]. The essential oil has been demonstrated to attenuate skin inflammation and exhibit both antioxidant and antigenotoxic activities [8,11]. Moreover, it has also exhibited antimicrobial activity against several pathogens, including the fungi *Malassezia furfur* and *Candida albicans*, bacteria such as *Staphylococcus epidermidis*, and mites like *Rhipicephalus linnaei* [9,12]. Geraniol has also demonstrated notable bioactive properties, exhibiting antimicrobial activity against several bacterial species, including *Escherichia coli*, *Helicobacter pylori*, *Haemophilus influenzae*, and *Streptococcus pneumoniae*. In addition to its antibacterial effects, geraniol shows broad antifungal activity, further supporting its potential as a multifunctional antimicrobial compound [13,14]. Beyond its antimicrobial potential, this compound has been reported to modulate key inflammatory and antioxidant signaling pathways, and it further displays significant antinociceptive activity [15,16]. Moreover, several industries, particularly the fragrance sector, use geraniol as a high-value scent compound, underscoring the relevance of its abundance in *C. martini* essential oil [7].

Antimicrobial resistance represents a rapidly expanding global challenge due to the ability of bacteria, fungi, viruses, or parasites to withstand antimicrobial treatment. In particular, antibiotic resistance specifically involves decreased bacterial susceptibility or complete resistance to antibiotic treatments. Several mechanisms are involved in this phenomenon, including enzymatic inactivation of the drug, reduced permeability, modification of the antimicrobial target, and enhanced efflux pump activity, which leads to high levels of drug expulsion [17,18]. It is estimated that bacterial infections account for approximately 7.7 million deaths annually, with about 4.95 million linked to drug-resistant pathogens and 1.27 million directly attributable to bacteria that no longer respond to currently available antibiotics [19]. Furthermore, several authors have anticipated that these mortality levels will continue to increase in the coming decades, reflecting the global burden of antimicrobial resistance [20]. The World Health Organization recognizes this issue as one of the top ten threats to global health. An updated version of the Bacterial Priority Pathogens List, highlighting antibiotic-resistant bacteria that pose a serious threat to global health, was released in 2024. Within this classification, *Staphylococcus aureus* and *Pseudomonas aeruginosa* are listed among the high-priority pathogens, reflecting their significant clinical burden, highlighting methicillin-resistant *Staphylococcus aureus* and carbapenem-resistant *Pseudomonas aeruginosa*. *Streptococcus* spp., including macrolide-resistant Group A *Streptococci*, macrolide-resistant *Streptococcus pneumoniae*, and penicillin-resistant Group B *Streptococci*, are categorized as medium-priority pathogens [21]. This underscores the urgent need for strengthened infection prevention strategies, continuous surveillance, and innovative antimicrobial development. In this context, natural products traditionally used for the treatment of infectious diseases have gained renewed scientific interest as potential sources of novel antimicrobial compounds.

Given the traditional use of *Cymbopogon* species in the management of colds, sore throats, and tracheitis [22], the objective of this work is to study the antimicrobial properties of *C. martini* essential oil on bacterial isolates form the respiratory tract. The impact on skin isolates was also assessed, given that the oil is also suitable for topical application and has anti-inflammatory properties. The effects of the product were contrasted with those of geraniol, its major constituent, also evaluating the impact of both samples on hepatic mammalian cells.

## 2. Materials and Methods

### 2.1. Essential Oil

**Figure 1:**
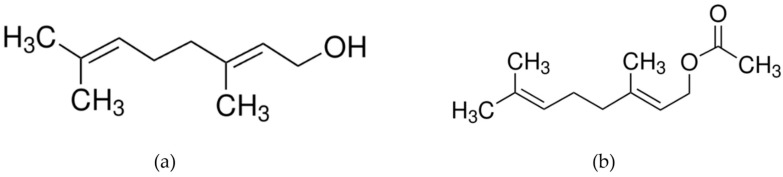
Molecular structure of (**a**) geraniol (Mw: 154.25 g/mol) and (**b**) geranyl acetate (Mw: 196.29 g/mol).

*C. martini* essential oil was provided by Pranarôm (Ghislenghien, Belgium), which also provided a description of its main characteristics. The essential oil was obtained by steam distillation; the batch was OF012293, obtained from the aerial parts of a *C. martini* var. motia (Roxb.) W. Watson cultivar from India. The essential oil was stored at room temperature and protected from light exposure. Stock solutions were prepared immediately before use. Gas chromatography–mass spectrometry (GC-MS) was also performed by the supplier under standardized conditions (Table 1). Chromatographic analyses were carried out using a TSQ92105012 instrument equipped with a 5SilMS 60-0.25-0.25 column. The temperature program consisted of an initial hold of 2 min at 40 °C, followed by a linear increase of 5 °C/min up to 320 °C, with a final hold for 10 min. Helium was employed as the carrier gas at a constant pressure of 3.2 bar. The method allowed for a detection limit of 0.01%, ensuring sensitive and reproducible quantification. The chemical structures of the main compounds identified in the essential oil can be seen in Figure 1. The chromatogram is presented in the Supplementary Materials.

### 2.2. Microorganisms Tested

Human clinical bacterial isolates were obtained from skin, soft tissues, and both upper and lower respiratory tracts. All the strains were isolated and identified at the Microbiology Laboratory of the Hospital Clínico Universitario Lozano Blesa. Identification was carried out with a MALDI Biotyper Sirius system (Bruker Daltonics, Bremen, Germany). Antibiograms that detail the antimicrobial resistance of clinical isolates are provided in the Supplementary Materials.

### 2.3. Antibacterial Activity

#### 2.3.1. Minimum Inhibitory Concentration (MIC)

The broth microdilution method with 96-well plates was used to determine the MIC for both the essential oil and geraniol purchased from Sigma-Aldrich (Merck KGaA, Darmstadt, Germany). A bacterial preculture was then incubated to 0.5 McFarland. From this suspension, 10 µL was added to a 96 well plate containing 190 µL of Tryptic Soy Broth (Thermo Fisher Scientific, Waltham, MA, USA) or Mueller–Hinton Broth supplemented with 3% lysed horse blood media (Beckman Coulter Inc., Brea, CA, USA), according to the bacterial requirements. Samples were previously dissolved in the media with dimethyl sulfoxide (Thermo Fisher Scientific, Waltham, MA, USA) as a cosolvent. Negative controls and solvent controls were also prepared. The plates were incubated overnight at 37 °C, and the MIC was defined as the lowest antimicrobial concentration at which no visible bacterial growth was observed, indicated by the absence of turbidity. Control plates were prepared to verify the bacterial concentration. Three independent replicates were included.

#### 2.3.2. Minimum Bactericidal Concentration (MBC)

To assess the MBC, aliquots from the wells were diluted in Tryptic Soy Broth or Mueller–Hinton Broth supplemented with 3% lysed horse blood and plated on Mueller–Hinton agar or chocolate agar (BioMérieux, Marcy-l’Étoile, France). The plates were incubated overnight, and colonies were counted to calculate the MBC, defined as the lowest concentration that resulted in a 99.9% reduction in bacterial growth. All experiments were carried out in triplicate. An MBC/MIC ratio ≤ 4 was used to define bactericidal activity, whereas an MBC/MIC ratio > 4 was considered indicative of bacteriostatic activity [23].

### 2.4. Cytotoxicity

HepG2 cells, originating from human hepatocellular carcinoma, were employed to assess the potential cytotoxic effects of *C. martini* essential oil. The cell line, obtained from the American Type Culture Collection (ATCC, Manassas, VA, USA), was maintained in Dulbecco’s Modified Eagle Medium obtained from Gibco (Thermo Fisher Scientific, Waltham, MA, USA) supplemented with 10% fetal bovine serum (Thermo Fisher Scientific, Waltham, MA, USA) and 1% penicillin–streptomycin (Thermo Fisher Scientific, Waltham, MA, USA). Cells were incubated at 37 °C in a humidified atmosphere containing 5% CO_2_.

Cytotoxicity was determined using the 3-(4,5-dimethylthiazol-2-yl)-2,5-diphenyltetrazolium bromide (MTT) assay. Briefly, 2 × 10^4^ cells were seeded per well in 96-well plates and allowed to adhere for 24 h. The medium was then replaced with Dulbecco’s Modified Eagle Medium containing the essential oil or geraniol at different concentrations. Control wells received only culture medium without extract. Cells were exposed to the treatment for 24 h.

Following incubation, 100 µL of MTT solution (0.4 mg/mL) was added to each well, and the plates were incubated for 2 h in the dark. After the formation of formazan crystals, the MTT solution was carefully removed, and the crystals were dissolved in dimethyl sulfoxide. The absorbance was measured at 550 nm using a Synergy H1 hybrid multimode reader (Biotek Instruments, Bad Friedrichshall, Germany). All experiments were carried at least in triplicate. Concentration of cytotoxicity 50% (CC_50_) was also determined.

### 2.5. Statistical Analysis

GraphPad Prism 10 was used to perform the statistical analysis. Grubbs’ test was utilized to identify significant outliers. To compare the different concentrations tested with the untreated conditions or controls, a one-way analysis of variance (ANOVA) with Dunnett’s post hoc test was performed. CC_50_ values were also calculated with GraphPad Prism 10 through a non-linear regression. The obtained MIC and MBC values for the bacterial isolated were analyzed descriptively.

## 3. Results

### 3.1. Antimicrobial Activity

Table 2 shows the antimicrobial potential of both the essential oil and geraniol against different bacteria isolates. For the Gram-positive bacteria, *C. martini* displayed MIC values ranging from 125 to 300 µg mL^−1^ and MBC values from 250 to 500 µg mL^−1^. Those isolates with a lower MIC were *Streptococcus agalactiae* and *Streptococcus anginosus*, both isolates having MICs of 125 µg mL^−1^ and an MBC of 250 µg mL^−1^, while the pure compound showed higher values. For both *Streptococcus pyogenes* isolates, the essential oil showed MIC and MBC values of 250 µg mL^−1^, while the values for geraniol could not be calculated. The MIC and MBC values of *C. martini* essential oil against both *Staphylococcus* species tested were 300 and 400 µg mL^−1^ respectively.

For the Gram-negative bacteria, higher MIC and MBC values were found for the oil and geraniol, from 250 and 300 µg mL^−1^, respectively, to values higher than 1000 µg mL^−1^ and therefore were not determined. The lowest MIC values were determined for *Moraxella catarrhalis* (*C. martini*, 250 µg mL^−1^; geraniol, 300 µg mL^−1^). None of the tested products were active against *Pseudomonas aeruginosa* isolates (MIC > 1000 µg mL^−1^). *Klebsiella oxytoca* and *Achromobacter xylosoxidans* were the only isolates where the MIC and MBC values were lower for geraniol (300 µg mL^−1^) than the essential oil (450 µg mL^−1^). For both *Escherichia coli* isolates, MIC values were lower for the essential oil (400 µg mL^−1^ surgical wound; 350 µg mL^−1^ tracheal aspirate) than geraniol (500 µg mL^−1^). As the MBC/MIC ratio was <4 for all strains showing activity, both the essential oil and geraniol were considered bactericidal for those isolates.

### 3.2. Impact on Mammalian Cells

**Figure 2:**
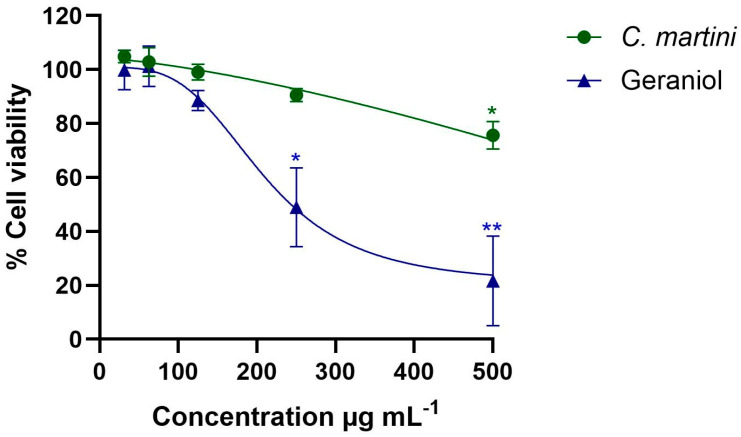
Impact of *C. martini* essential and geraniol on HepG2 cells after 24 h of exposure to different concentrations (from 31.25 µg mL^−1^ to 500 µg mL^−1^) using the MTT assay. All data were normalized relative to the untreated control group. Data are presented as mean ± SEM from three independent experiments, each performed in triplicate. Statistical significance was determined by one-way ANOVA (statistical significance: * *p* < 0.05; ** *p* < 0.01). A higher concentration range (62.5 to 1000 µg mL^−1^) than the represented one was used to determine the CC_50_ values. The CC_50_ values were obtained by regression analysis following a non-linear curve fitting. *C. martini* essential oil CC_50_ = 761 ± 69 µg mL^−1^. Geraniol CC_50_ = 205 ± 8 µg mL^−1^.

The impact of both the essential oil and geraniol in the range of concentrations from 62.5 to 500 µg mL^−1^ on mammalian cells is presented in Figure 2. *C. martini* essential oil significantly reduced the viability of HepG2 cells at the highest concentration tested, 500 µg mL^−1^ (75.64% viability; *p* < 0.05). Geraniol also significantly reduced the viability at 250 µg mL^−1^ and 500 µg mL^−1^. The essential oil showed a higher CC_50_ value (761 ± 69 µg mL^−1^), than the pure compound tested under the same conditions (205 ± 8 µg mL^−1^)

## 4. Discussion

*C. martini* essential oil holds considerable commercial relevance across multiple industries, including the perfumery, cosmetics, agri-food and pharmaceutical industries, due to its persistent, rose-like fragrance that enhances product quality and consumer acceptance [7]. Additionally, it is also considered a valuable product due to its high geraniol content. In palmarosa oil, geraniol is the main component, representing 82.83% of the volatile fraction. Other compounds found were geranyl acetate (7.21%), linalool (7.71), and beta-caryophyllene (1.89%) (Table 1). This composition aligns with those in previous reports [7,24,25].

Geraniol is a monoterpenic alcohol employed in commercial formulations, particularly within the cosmetic and household product industries. This compound is a naturally occurring constituent of numerous essential oils such as *Thymus daenensis*, *Aframomum citratum*, *Elettariopsis elan* or those of the genus *Cymbopogon* [14]. In addition, recent reports have highlighted its antimicrobial properties, aligning with the results of this work (Table 2) [26]. Table 2 shows the antimicrobial potential of both the oil and geraniol against different bacteria isolates. Surprisingly, the oil showed potential against the genus *Streptococcus*, with an MIC value of 125 µg mL^−1^ for both *Streptococcus agalactiae* and *Streptococcus anginosus* and 250 µg mL^−1^ for *Streptococcus dysgalactiae* and the two isolates of *Streptococcus pyogenes*. This genus includes over a hundred microbial species that colonize mucosal surfaces in humans and animals. *Streptococcus pyogenes* alone causes around 700 million infections each year, while *Streptococcus agalactiae* is associated with miscarriages, preterm births, and severe neonatal infections such as sepsis, pneumonia, and meningitis [27]. *Streptococcus anginosus*, usually a commensal of the oral, gastrointestinal, and genitourinary tracts, is often involved in abscesses and other purulent infections, and more recent research has implicated this species in gastric carcinogenesis through chronic gastric inflammation and progression to cancer [28]. To the best of our knowledge, this is the first report of the effects of *C. martini* essential oil on these bacteria. For geraniol, the results align with a previous report that studied the potential of this pure compound against *Streptococcus pyogenes* [29]. Both the oil and geraniol were also active against *Staphylococcus aureus*, aligning with previous reports [29,30].

*Escherichia coli* is a Gram-negative bacterium that constitutes part of the normal intestinal microbiota. However, under certain conditions, it can behave as an opportunistic pathogen, capable of causing severe infections beyond the gastrointestinal tract. In the present study, the isolates were obtained from tracheal aspirates and surgical wound samples. In cutaneous wounds, *Escherichia coli* may lead to skin and soft tissue infections that can progress and become life-threatening, particularly in patients who are immunocompromised [31,32]. The detection of *Escherichia coli* in tracheal aspirates is also significant, as this organism can act as an etiological agent of lower respiratory tract infections, including ventilator-associated pneumonia. In addition, such isolates often exhibit multidrug resistance and complex virulence profiles, posing major therapeutic challenges [33,34]. In this work, the oil and geraniol showed effects on *Escherichia coli*, with MIC values of 400 µg mL^−1^ and 350 µg mL^−1^ for the surgical wound and tracheal aspirate samples, respectively.

*C. martini* was also active against other Gram-negative bacteria relevant in clinical settings, namely, *Morganella morganii* (MIC 350 µg mL^−1^; MBC 500 µg mL^−1^), *Moraxella catarrhalis* (MIC 250 µg mL^−1^; MBC 250 µg mL^−1^), *Serratia marcescens* (MIC 350 µg mL^−1^; MBC 400 µg mL^−1^), and *Klebsiella oxytoca* (MIC 450 µg mL^−1^; MBC 450 µg mL^−1^). However, neither of the samples showed activity against *Pseudomonas aeruginosa*, with MIC values higher than 1000 µg mL^−1^ and therefore were not determined.

Geraniol and palmarosa essential oil seem to exert their antibacterial activity primarily by disrupting bacterial cell membrane integrity. Geraniol has been described as inducing membrane disintegration, causing ion leakage and altered fatty acid composition, which compromise membrane fluidity and leads to cell death [35,36]. Palmarosa oil may additionally generate reactive oxygen species and inhibit bacterial efflux pumps, further enhancing antimicrobial activity [12]. Overall, their activity may reflect a multifaceted mode of action primarily centered on membrane disruption. Additionally, it is worth noting that in most cases the essential oil displayed lower MIC values than those of geraniol (Table 2). This could be a result of the synergistic effect of the different compounds found in the oil (Table 1) since geranyl acetate, linalool, and beta-caryophyllene have demonstrated antimicrobial effects in other works [37,38,39]. Both the essential oil and geraniol reduced the viability of the HepG2 cells at the highest concentrations (Figure 2); however, the essential oil displayed a broader safety range for mammalian cells while, as mentioned, also displaying lower MIC values.

## 5. Conclusions

Plants from the *Cymbopogon* genus have a long history in traditional medicine. In this work, the essential oil obtained from the aerial parts of *C. martini* as well as its main constituent, geraniol, showed potential activity on clinical isolates from the skin and respiratory tract, being particularly strong against *Streptococcus* spp. and *Staphylococcus* spp. For most of the clinical bacteria tested, the essential oil showed lower MIC values and therefore higher antibacterial potency. This seems to indicate that the potential of other compounds found in the essential oil such as geranyl acetate, linalool or beta-caryophyllene cannot be overlooked. Additionally, the essential oil was less cytotoxic to HepG2 cells when compared with the pure compound. These results highlight the potential of *C. martini* for pharmaceutical uses and applications, particularly as an antimicrobial.

## Figures and Tables

**Table 1:** Phytochemical profile of *C. martini* essential oil provided by the supplier.

Phytochemical Profile				
Peak	Retention Time	Constituent	Cas Number	%
1	15.56	6-methyl-5-hepten-2-one	110-93-3	0.05
2	15.70	Beta-myrcene	123-35-3	0.19
3	16.26	Alpha-phellandrene	99-83-2	0.01
4	17.07	Limonene	138-86-3	0.10
5	17.13	Cis-beta-ocimene	3338-55-4	0.39
6	17.48	Trans-beta-ocimene	3779-61-1	1.51
7	19.15	Linalool	78-70-6	2.71
8	22.88	Nerol	106-25-2	0.21
9	23.30	Neral	106-26-3	0.13
10	23.85	Geraniol	106-24-1	82.83
11	24.18	Geranial	141-27-5	0.25
12	24.90	Neryl formate	2142-94-1	0.06
13	26.60	Neryl acetate	141-12-8	0.02
14	27.04	Geranyl acetate	105-87-3	7.21
15	27.37	Ylangene	14912-44-8	0.01
16	27.57	Beta-elemene	141-12-8	0.09
17	28.52	Beta-caryophyllene	87-44-5	1.89
18	29.42	Alpha-humulene	6753-98-6	0.11
19	31.46	Geranyl butyrate	106-29-6	0.16
20	31.67	Trans-nerolidol	40716-66-3	0.12
21	32.59	Caryophyllene oxide	1139-30-6	0.19
22	35.23	Farnesol	4602-84-0	0.82
23	35.87	Geranyl caproate	4602-84-0	0.62
24	37.66	Farnesyl acetate	4128-17-0	0.07
25	39.90	Geranyl caprylate	51532-26-4	0.13
**Total**				**99.88**
**Classification of identified compounds**				
**Terpene class**			**Percentage (%)**	
**Monoterpene hydrocarbons:** β-myrcene, α-phellandrene, limonene, cis-β-ocimene, trans-β-ocimene			2.20	
**Oxygenated monoterpenoids:** linalool, nerol, neral, geraniol, geranial, neryl formate, neryl acetate, geranyl acetate, geranyl butyrate, geranyl caproate, geranyl caprylate			94.33	
**Oxygenated sesquiterpenoids:** trans-nerolidol, caryophyllene oxide, farnesol, farnesyl acetate			1.20	
**Others:** 6-methyl-5-hepten-2-one			0.05	
**Total identified compounds**			**99.88**	

**Table 2:** Antibacterial effect of palmarosa (*C. martini*) essential oil and geraniol expressed as MIC and MBC (µg mL^−1^) values on clinical isolates. All experiments were conducted in triplicate, with each assay comprising at least three technical replicates. Reported values correspond to the mean of the obtained results. Standard deviation (SD) is not shown in the table for MIC and MBC values. because these endpoints were determined in discrete concentration steps, and identical values were obtained across replicates.

Antibacterial Activity					
Bacterial Strain	Isolated From	*C. martini*		Geraniol	
		MIC	MBC	MIC	MBC
Gram-positive					
*Streptococcus agalactiae*	Diabetic foot ulcer	125	250	300	400
*Streptococcus anginosus*	Surgical wound	125	250	500	500
*Streptococcus dysgalactiae*	Otic swab	250	500	300	400
*Streptococcus pyogenes*	Pharyngeal sample	250	250	>1000	>1000
*Streptococcus pyogenes*	Otic swab	250	250	>1000	>1000
*Staphylococcus aureus*	Non-surgical wound	300	400	500	600
*Staphylococcus lugdunensis*	Otic swab	300	400	500	500
Gram-negative					
*Pseudomonas aeruginosa*	Ulcer	>1000	>1000	>1000	>1000
*Pseudomonas aeruginosa*	Non-surgical wound	>1000	>1000	>1000	>1000
*Pseudomonas aeruginosa*	Sputum	>1000	>1000	>1000	>1000
*Morganella morganii*	Non-surgical wound	350	500	500	500
*Escherichia coli*	Surgical wound	400	500	500	550
*Escherichia coli*	Tracheal aspirate	350	450	500	500
*Moraxella catarrhalis*	Otic swab	250	250	300	300
*Achromobacter xylosoxidans*	Otic swab	900	>1000	500	500
*Serratia marcescens*	Sputum	350	400	500	500
*Klebsiella oxytoca*	Sputum	450	450	300	300

## Data Availability

The original contributions presented in this study are included in this article/Supplementary Materials. Further inquiries can be directed to the corresponding author(s).

## Supplementary Materials

The following supporting information can be downloaded at: https://www.mdpi.com/article/10.3390/pharmaceutics18010039/s1, Table S1: Antibiogram of *Streptococcus agalactiae* (Group B) isolated from a diabetic foot ulcer; Table S2: Antibiogram of *Streptococcus anginosus* isolated from a surgical wound; Table S3: Antibiogram of *Streptococcus pyogenes* isolated from a pharyngeal sample; Table S4: Antibiogram of *Streptococcus pyogenes* isolated from a pharyngeal sample; Table S5: Antibiogram of *Staphylococcus aureus* isolated from a non-surgical wound; Table S6: Antibiogram of *Pseudomonas aeruginosa* isolated from an ulcer; Table S7: Antibiogram of *Pseudomonas aeruginosa* isolated from a non-surgical wound; Table S8: Antibiogram of *Pseudomonas aeruginosa* isolated from a sputum; Table S9: Antibiogram of *Morganella morganii* isolated from a non-surgical wound; Table S10: Antibiogram of *Escherichia coli* isolated from a surgical wound; Table S11: Antibiogram of *Escherichia coli* isolated from a tracheal aspirate; Table S12: Antibiogram of *Moraxella catarrhalis* isolated from an otic swab; Table S13: Antibiogram of *Moraxella catarrhalis* isolated from an otic swab; Table S14: Antibiogram of *Serratia marcescens* isolated from a sputum; Table S15: Antibiogram of *Klebsiella oxytoca* isolated from a sputum; Figure S1: Chromatogram of *C. martini* EO analyzed by GC-MS.

## Abbreviations

The following abbreviations are used in this manuscript:

*C. martini*	*Cymbopogon martini*
MTT	3-(4,5-dimethylthiazol-2-yl)-2,5-diphenyltetrazolium bromide
