# Supplementary material for: Antibacterial Activity of Palmarosa (Cymbopogon martini (Roxb.) Will.Watson) Essential Oil and Geraniol Against Clinical Isolates from Respiratory, Skin, and Soft Tissue Infections
Source: Pharmaceutics. 2025 Dec 27;18(1):39. doi: 10.3390/pharmaceutics18010039 (PMC12844656; doi:10.3390/pharmaceutics18010039)
Supplement: Supplementary file 1 [file pharmaceutics-18-00039-s001.zip › pharmaceutics-4041467-supplementary.pdf]

Supplementary Material 1: Clinical Sources and Antibiotic Susceptibility Profiles of Bacterial Isolates Used in This Study

Table S1: Antibiogram of *Streptococcus agalactiae* (Group B) isolated from a diabetic foot ulcer.

<table>
<tr><th colspan="2">Streptococcus agalactiae (Group B) – Isolated from a diabetic foot ulcer</th></tr>
<tr><th>Antibiotic</th><th>Result</th></tr>
<tr><td>Penicillin</td><td>Susceptible</td></tr>
<tr><td>Oxacillin</td><td>—</td></tr>
<tr><td>Vancomycin</td><td>Susceptible</td></tr>
<tr><td>Teicoplanin</td><td>—</td></tr>
<tr><td>Daptomycin</td><td>—</td></tr>
<tr><td>Linezolid</td><td>—</td></tr>
<tr><td>Erythromycin</td><td rowspan="2">Resistant</td></tr>
<tr><td>Clindamycin</td></tr>
<tr><td>Tetracycline</td><td>—</td></tr>
<tr><td>Trimethoprim/Sulfamethoxazole</td><td>—</td></tr>
<tr><td>Levofloxacin</td><td>—</td></tr>
</table>

Table S2: Antibiogram of *Streptococcus anginosus* isolated from a surgical wound.

***Streptococcus anginosus* – Isolated from a Surgical wound**	
**Antibiotic**	**Result**
Penicillin	Susceptible
Ampicillin	Susceptible
Amoxicillin/Clavulanic acid	—
Ceftriaxone	Susceptible
Cefepime	—
Ertapenem	—
Imipenem	—
Meropenem	—
Vancomycin	Susceptible

Erythromycin	—
Clindamycin	Susceptible
Gentamicin	—
Tobramycin	—
Trimethoprim/Sulfamethoxazole	—
Ciprofloxacin	—

Table S3: Antibiogram of *Streptococcus pyogenes* isolated from a pharyngeal sample.

***Streptococcus pyogenes* (Group A) – Isolated from a pharyngeal sample**	
**Antibiotic**	**Result**
Penicillin	Susceptible
Erythromycin	Susceptible
Clindamycin	Susceptible

Table S4: Antibiogram of *Streptococcus pyogenes* isolated from a pharyngeal sample.

***Streptococcus pyogenes* (Group A) – Isolated from an otic sample**	
**Antibiotic**	**Result**
Penicillin	Susceptible
Piperacillin/Tazobactam	—
Meropenem	—
Erythromycin	Susceptible
Clindamycin	Susceptible
Trimethoprim/Sulfamethoxazole	—

Table S5: Antibiogram of *Staphylococcus aureus* isolated from a non-surgical wound.

***Staphylococcus aureus*– Isolated from a Non-surgical wound**	
**Antibiotic**	**Result**
Piperacillin/Tazobactam	—
Ceftazidime	—
Cefepime	—

Imipenem	—
Meropenem	—
Oxacillin	Susceptible
Vancomycin	Susceptible
Teicoplanin	Susceptible
Daptomycin	Susceptible
Linezolid	Susceptible
Erythromycin	Susceptible
Clindamycin	Susceptible
Tetracycline	Susceptible
Tobramycin	—
Amikacin	—
Trimethoprim/Sulfamethoxazole	Susceptible
Ciprofloxacin	Susceptible, increased exposure
Levofloxacin	Susceptible, increased exposure

Table S6: Antibiogram of *Pseudomonas aeruginosa* isolated from an ulcer.

***Pseudomonas aeruginosa* – Isolated from an ulcer**	
**Antibiotic**	**Result**
Piperacillin/Tazobactam	Susceptible, increased exposure
Ceftazidime	Susceptible, increased exposure
Cefepime	Susceptible, increased exposure
Imipenem	Susceptible, increased exposure
Meropenem	Susceptible
Oxacillin	—
Vancomycin	—
Teicoplanin	—
Daptomycin	—

Linezolid	—
Erythromycin	—
Clindamycin	
Tetracycline	—
Tobramycin	Resistant
Amikacin	Resistant
Trimethoprim/Sulfamethoxazole	—
Ciprofloxacin	Resistant
Levofloxacin	—

Table S7: Antibiogram of *Pseudomonas aeruginosa* isolated from a non-surgical wound.

***Pseudomonas aeruginosa* – Isolated from a non-surgical wound**	
**Antibiotic**	**Result**
Piperacillin/Tazobactam	Susceptible, increased exposure
Ceftazidime	Susceptible, increased exposure
Cefepime	Susceptible, increased exposure
Tobramycin	Susceptible
Ciprofloxacin	Resistant

Table S8: Antibiogram of *Pseudomonas aeruginosa* isolated from a sputum.

***Pseudomonas aeruginosa* – Isolated from a sputum**	
**Antibiotic**	**Result**
Penicillin	—
Amoxicillin/Clavulanic acid	—
Piperacillin/Tazobactam	Susceptible

Ceftazidime	Susceptible
Cefepime	Susceptible
Aztreonam	Susceptible
Imipenem	Resistant
Meropenem	Resistant
Oxacillin	—
Vancomycin	—
Teicoplanin	—
Daptomycin	—
Linezolid	—
Erythromycin	—
Clindamycin	—
Tetracycline	—
Tobramycin	Susceptible
Amikacin	Susceptible
Trimethoprim/Sulfamethoxazole	—
Colistin	Susceptible
Ciprofloxacin	Susceptible
Levofloxacin	Susceptible

Table S9: Antibiogram of *Morganella morganii* isolated from a non-surgical wound.

***Morganella morganii*– Isolated from a non-surgical wound**	
**Antibiotic**	**Result**
Ampicillin	Resistant
Amoxicillin/Clavulanic acid	Resistant
Oxacillin	—
Erythromycin	—
Clindamycin	—
Tetracycline	—

Gentamicin	Susceptible
Tobramycin	Susceptible
Trimethoprim/Sulfamethoxazole	Susceptible
Ciprofloxacin	Susceptible
Levofloxacin	Susceptible

Table S10: Antibiogram of *Escherichia coli* isolated from a surgical wound.

***Escherichia coli*– Isolated from a surgical wound**	
**Antibiotic**	**Result**
Ampicillin	Resistant
Amoxicillin/Clavulanic acid	Resistant
Piperacillin/Tazobactam	Resistant
Cefotaxime	Susceptible
Cefepime	Susceptible, increased exposure
Gentamicin	Susceptible
Tobramycin	Susceptible
Trimethoprim/Sulfamethoxazole	Susceptible
Ciprofloxacin	Susceptible

Table S11: Antibiogram of *Escherichia coli* isolated from a tracheal aspirate.

***Escherichia coli*– Isolated from a tracheal aspirate**	
**Antibiotic**	**Result**
Ampicillin	Resistant
Amoxicillin/Clavulanic acid	Resistant
Piperacillin/Tazobactam	Susceptible
Cefuroxime	Resistant
Cefotaxime	Resistant
Ceftazidime	Resistant
Cefepime	Resistant

Aztreonam	Resistant
Ertapenem	Susceptible
Imipenem	Susceptible
Meropenem	Susceptible
Gentamicin	Susceptible
Tobramycin	Resistant
Amikacin	Susceptible
Trimethoprim/Sulfamethoxazole	Resistant
Ciprofloxacin	Resistant
Levofloxacin	Resistant

Table S12: Antibiogram of *Moraxella catarrhalis* isolated from an otic swab.

***Moraxella catarrhalis* – Isolated from an otic swab**	
**Antibiotic**	**Result**
Penicillin	—
Amoxicillin/Clavulanic acid	Susceptible
Oxacillin	—
Vancomycin	—
Erythromycin	Susceptible
Clindamycin	—
Tetracycline	—
Trimethoprim/Sulfamethoxazole	Susceptible
Ciprofloxacin	—
Levofloxacin	Susceptible

Table S13: Antibiogram of *Moraxella catarrhalis* isolated from an otic swab.

***Achromobacter xylosoxidans* – Isolated from an otic swab**	
**Antibiotic**	**Result**
Penicillin	—

Piperacillin/Tazobactam	Susceptible
Meropenem	Susceptible
Erythromycin	—
Clindamycin	—
Trimethoprim/Sulfamethoxazole	Susceptible

Table S14: Antibiogram of *Serratia marcescens* isolated from a sputum.

***Serratia marcescens* – Isolated from a Sputum**	
**Antibiotic**	**Result**
Ampicillin	Resistant
Amoxicillin/Clavulanic acid	Resistant
Cefuroxime	—
Cefoxitin	Resistant
Gentamicin	Susceptible
Trimethoprim/Sulfamethoxazole	Susceptible
Ciprofloxacin	Susceptible
Levofloxacin	Susceptible

Table S15: Antibiogram of *Klebsiella oxytoca* isolated from a sputum.

***Klebsiella oxytoca* – Isolated from a Sputum**	
**Antibiotic**	**Result**
Ampicillin	Resistant
Amoxicillin/Clavulanic acid	Susceptible
Cefuroxime	Susceptible, increased exposure
Cefoxitin	—
Gentamicin	Susceptible
Trimethoprim/Sulfamethoxazole	Susceptible
Ciprofloxacin	Susceptible
Levofloxacin	Susceptible

Resistance patterns were determined by the hospital's microbiology laboratory using standard procedures. Some bacterial isolates, such as *Staphylococcus lugdunensis*, were susceptible to all tested antibiotics and therefore were not included in the antibiogram tables.

Supplementary Material 2: Chemical profile of *C. martini* essential oil provided by Pranarôm (Ghislenghien, Belgium)

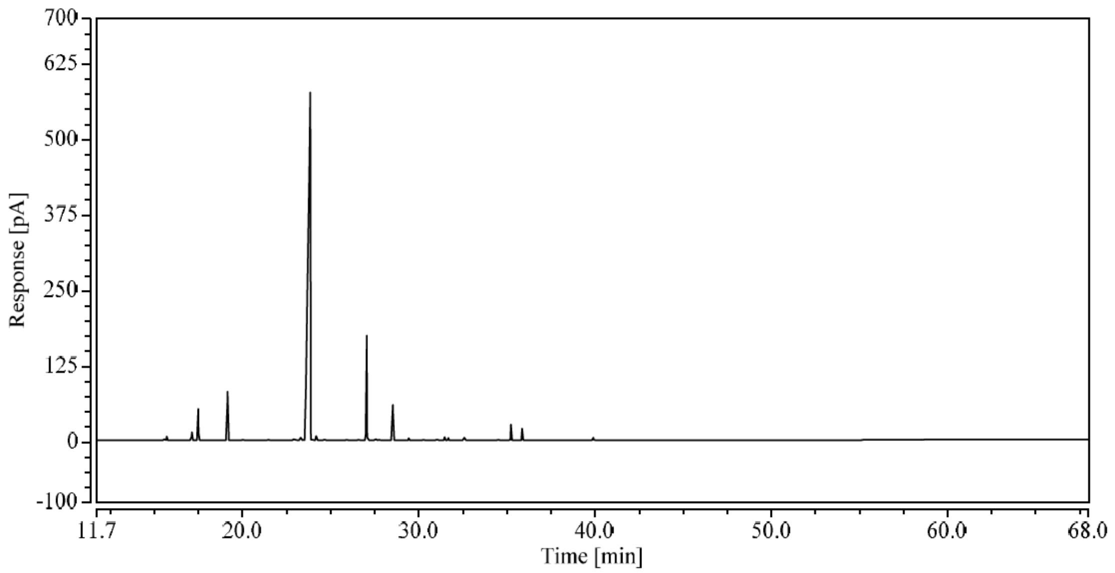

Figure S1: Chromatogram of *C. martini* essential oil analysed by GC-MS.
